# Localized, time-dependent responses of rat cranial bone to repeated mild traumatic brain injuries

**DOI:** 10.1038/s41598-022-18643-5

**Published:** 2022-09-01

**Authors:** Larissa K. Dill, Natalie A. Sims, Ali Shad, Chidozie Anyaegbu, Andrew Warnock, Yilin Mao, Melinda Fitzgerald, Bridgette D. Semple

**Affiliations:** 1 Department of Neuroscience, Central Clinical School, Monash University, Level 6, The Alfred Centre, 99 Commercial Road, Melbourne, VIC 3004 Australia; 2 Alfred Health, Prahran, VIC Australia; 3 Ear Science Institute Australia, Subiaco, WA Australia; 4 Curtin Health Innovation Research Institute, Faculty of Health Sciences, Bentley, WA Australia; 5 St Vincent’s Institute of Medical Research, Melbourne, VIC Australia; 6 Department of Medicine (St. Vincent’s Health Melbourne), The University of Melbourne, Fitzroy, VIC Australia; 7 The Perron Institute for Neurological and Translational Science, Sarich Neuroscience Research Institute Building, Nedlands, WA Australia; 8 Department of Medicine (Royal Melbourne Hospital), The University of Melbourne, Parkville, VIC Australia

**Keywords:** Brain injuries, Trauma, Bone

## Abstract

While it is well-established that bone responds dynamically to mechanical loading, the effects of mild traumatic brain injury (mTBI) on cranial bone composition are unclear. We hypothesized that repeated mTBI (rmTBI) would change the microstructure of cranial bones, without gross skull fractures. To address this, young adult female Piebald Viral Glaxo rats received sham, 1×, 2× or 3× closed-head mTBIs delivered at 24 h intervals, using a weight-drop device custom-built for reproducible impact. Skull bones were collected at 2 or 10 weeks after the final injury/sham procedure, imaged by micro computed tomography and analyzed at predetermined regions of interest. In the interparietal bone, proximal to the injury site, modest increases in bone thickness were observed at 2 weeks, particularly following 2× and 3× mTBI. By 10 weeks, 2× mTBI induced a robust increase in the volume and thickness of the interparietal bone, alongside a corresponding decrease in the volume of marrow cavities in the diploë region. In contrast, neither parietal nor frontal skull samples were affected by rmTBI. Our findings demonstrate time- and location-dependent effects of rmTBI on cranial bone structure, highlighting a need to consider microstructural alterations to cranial bone when assessing the consequences of rmTBI.

## Introduction

It is increasingly recognized that repeated head impacts can have detrimental consequences on brain function. Mild traumatic brain injuries (mTBIs), including concussions, are the most common form of brain injury, and accumulating evidence suggests that repeated mTBIs (rmTBI) may result in persistent symptomology and chronic neuropathological effects, associated with a range of neuropsychiatric and neurodegenerative disorders^1,2^. However, little consideration has been afforded to the direct or indirect effects of rmTBI on the cranial skull bones that protect the brain. Such knowledge is critical for understanding how the meninges, cerebral vasculature, lymphatics system and brain parenchyma also respond to a single injury, as well as any subsequent injuries^3,4^.

Bone is a dynamic living tissue with a high capacity for remodeling in response to mechanical forces as well as environmental factors, such as hormonal influences or changes in gravity^5^. Mechanical loading—induced by high-intensity exercise, for example, or trauma—is a well-established regulator of long bone mass, density and composition^6–8^. However, how this phenomenon applies in the context of head impacts to skull bones has been poorly studied to date.

Cranial bone is a three-layered sandwich-like structure comprised of outer layers of compact cortical bone surrounding a central layer of irregular porous bone. This central diploë region is highly variable and responsive to applied mechanical forces^9^. Significant damage to the cranium from a moderate or severe TBI, resulting in a skull fracture, has been associated with a greater degree of neuroinflammation and poorer functional outcomes in experimental TBI models^10,11^. Similarly, the occurrence of a skull fracture in patients with severe TBI is a predictor of in-hospital mortality as well as unfavorable outcomes by 6 months post-injury^12–14^. On the milder end of the injury spectrum, in the absence of skull fracture, mechanical loading may also influence bone structure, particularly in the context of repetitive impacts such as those sustained during competitive sporting activities.

Several recent studies have demonstrated that a TBI can promote bone formation, such as the development of neurogenic heterotopic ossification even in remote skeletal locations^15–17^. Case studies have also reported that the skull periosteum, the membranous tissue that covers the bone surfaces, has considerable potential for osteogenesis in response to local hematomas^18,19^. These findings suggest a complex relationship between bone and brain injury that likely extends beyond responsiveness to the direct mechanical forces applied by a head impact.

Preclinical studies have the potential to allow better understanding of the relationship between TBI and bone remodeling post-injury. In adolescent mice, we have previously reported that a single mTBI led to increased bone volume and strength of the impacted parietal bone by 5 weeks later, which may account for a reduced incidence of skull fractures when animals were exposed to a second mTBI impact in adulthood^11^. Subsequently, another study found that mTBI in adult mice led to decreased bone porosity in the contralateral (uninjured) hemisphere, which appeared to be mediated by the cannabinoid-1 receptor^20^. Together, this scant evidence drove the formation of our current aim: to evaluate whether rmTBI influences skull bone composition and structure.

A reproducible closed-head injury model was used to mimic single or rmTBI, as previously described^21,22^. This model generates a mild brain injury in the absence of macroscopic brain damage or overt skull fractures. Previous work with this paradigm has demonstrated acute oxidative damage after rmTBI spaced 24 h apart, particularly after 2× mTBI, which worsens over weeks and months post-injury alongside microglial reactivity and indications of myelin pathology^22,23^. Here, we turned our attention to the effects of head injury on the skull bones, hypothesizing that rmTBI would result in greater effects compared to a single mTBI, and those effects would develop in a time- and location-dependent manner.

## Materials and methods

### Animals and ethics

Young adult female Piebald Viral Glaxo rats (12 weeks of age; 160–200 g) were sourced from the Animal Resource Center (Murdoch, WA, Australia). Animals were housed in standard cages under specific pathogen-free conditions and a 12:12 h light–dark cycle, with food and water available ad libitum*,* and acclimatized to housing conditions for at least one week prior to experiments. All procedures were approved by The University of Western Australia Animal Ethics Committee (#RA/3/100/1366) and conducted according to the Australian Code of Practice for the Care and Use of Animals for Scientific Purposes from the National Health and Medical Research Council (NHMRC) and in compliance with the ARRIVE guidelines.

### Experimental mTBI model

A reproducible closed-head injury model was used to mimic mild, repeated TBI, as previously described^21,22^. In brief, rats were anesthetized with inhalant 4% isoflurane (4 L/min in oxygen) and maintained at 2% isoflurane (2 L/min oxygen), then positioned prone on a suspended Kimwipe (Kimberley-Clark, Irving, TX, USA). Using a custom-built weight drop device (Northeast Biomedical Inc., MA, USA)^22–24^, injury was induced by release—down a guide tube—of a 250 g weight from 1 m height, to impact the head at the midline, 2–3 mm anterior to the front of the ears (i.e. aligned with Lambda on the underlying skull). Upon impact, the rat fell through the Kimwipe onto the foam mat below, allowing for rotational forces to be sustained, consistent with mechanisms common in human mTBI^25–27^. Carprofen at 4 mg/kg i.p. (Norbrook Laboratories, Tullamarine, VIC, Australia) was administered post-procedure to all animals for analgesia. Rats were then allowed to recover on a heat pad at 37 °C until ambulatory.

**Figure 1:**
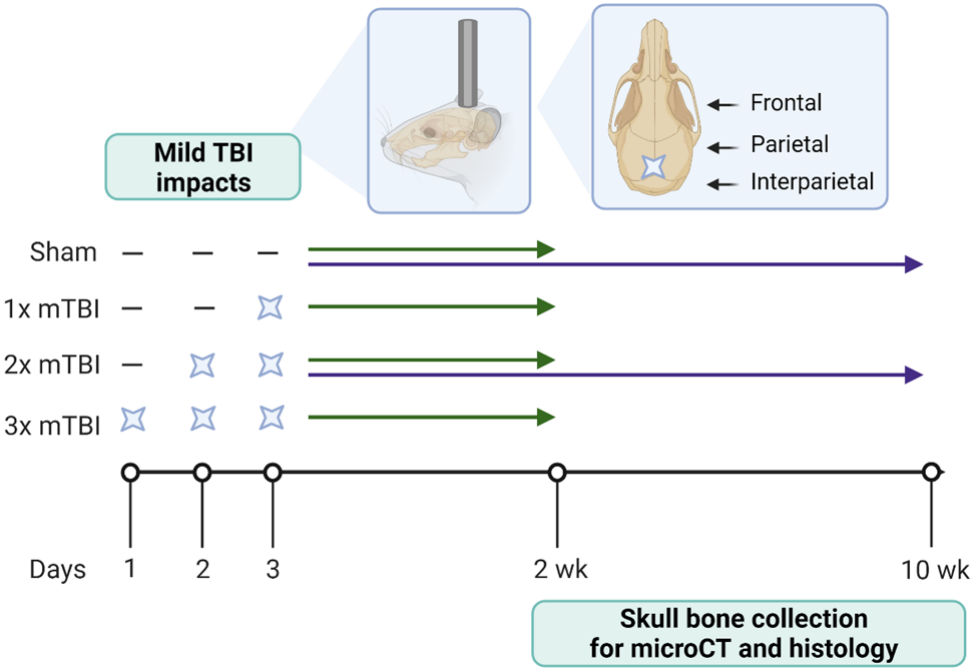
Schematic of study timeline. Rats received 0–3× mTBI impacts or sham procedures spaced 24 h apart. Animals were euthanized at either 2 weeks (sham, 1× mTBI, 2× mTBI and 3× mTBI groups) or 10 weeks (sham and 2× mTBI groups), with the time point referring to time following the last mTBI/sham procedure for collection of skull bones for microCT and histology. Illustrations depict the site of impact of the weight drop model on the rat skull, from a sagittal and dorsal view, respectively. Time points were chosen based upon previous findings in this model, whereby 2× mTBI animals often showed the greatest effects compared to sham controls^22,23^. Created with Biorender.com.

A total of 37 animals were randomized to one of four experimental groups, receiving either the sham procedure, 1×, 2× or 3× mTBI impacts, delivered at 24 h intervals. The 24 h inter-injury interval was chosen to approximate weekly exposure to sports-related rmTBI, based on evidence that biochemical processes such as basal metabolism occur around 6 times faster in rats than in humans^28^. Importantly, rats that received 1 or 2 mTBIs underwent the sham procedure when mTBI was not administered, to ensure equivalent anesthesia exposure for all animals. The sham procedure was identical to that described above, except for the weight drop impact itself (Fig. 1).

### Sample collection

At 2 weeks following the final injury/sham procedure, a subset of animals was euthanized with 100 mg/kg i.p. sodium pentobarbital (Lethobarb, Virbac, Australia) and perfused transcardially with sterile saline (0.9% sodium chloride) followed by 4% paraformaldehyde in phosphate buffer (0.1 M, pH 7.2). Cranial bone samples were dissected and collected from rats from the following experimental groups: Sham, n = 10; 1× mTBI, n = 7; 2× mTBI, n = 5; and 3× mTBI, n = 5.

At 10 weeks after the final injury/sham procedure, cranial bones were collected from two groups: Sham (n = 5) and 2× mTBI (n = 5). The 2× mTBI group was chosen since previous findings from this model indicated that the 2× mTBI resulted in the greatest injury response, i.e. a higher degree of oxidative stress, microglial reactivity, and myelin pathology compared to a single mTBI or even the 3× mTBI group^22,23^.

Collected samples were fixed in 4% paraformaldehyde then transferred into 70% ethanol for transport and storage.

### Micro computed tomography (microCT)

The rat cranial samples were imaged using a Siemens Inveon PET-SPECT-CT small animal scanner in micro-CT mode, at 80 kV and 500 uA with an exposure time of 600 ms (Monash Biomedical Imaging, Clayton, VIC Australia). Scans captured isotropic voxels of 9.7 µm in a field of approximately 1350 × 1350 × 1800 voxels.

Image stacks were reconstructed with x, y and z slice depth dimensions of 1 vx. ImageJ (https://imagej.nih.gov/ij/; National Institutes of Health, Bethesda, MD, USA) and the plugin BoneJ2^29^ were used on the high performance computing system MASSIVE^30^ to reconstruct, orient, crop and analyze the image stacks. All analyses were performed by an investigator blinded to experimental group.

Regions of interest (ROI) were defined relative to the midline and Lambda or Bregma, measured in voxels (Table 1). Interparietal and parietal bone ROI stacks consisted of 150 images, measuring 200 vx wide. Frontal bone ROI stacks consisted of 100 images, measuring 200 vx across. Bone and total tissue (including marrow space) was isolated using the statistical region merging segmentation^31^ with the Otsu threshold algorithm^32^ (thresholding settings described in Supplementary Methods). The BoneJ2 plugin was then used to measure bone volume in each sample, as well as the total tissue or object volume (i.e. bone plus marrow space) (see Supplementary Methods). The binary bone reconstructions were additionally used to calculate mean thickness of bone (averaged across 150 stack slices) and external surface area using BoneJ2.

**Table 1:** Region of interest (ROI) dimensions and loci.

ROI	Dimensions X, Y, Z (vx)	Dimensions (mm)	Position relative to midsagittal line	Position relative to Lambda or Bregma
Interparietal	200, 150, 150	1.96 W × 1.47 D	− 100 to + 100 vx; − 0.97 to + 0.97 mm	+ 100 to + 250 vx; + 0.97 to + 2.43 mm Lambda
Parietal	200, 150, 150	1.96 W × 1.47 D	− 350 to − 150 vx; − 3.39 to − 1.46 mm	− 350 to − 200 vx; − 3.39 to − 1.96 mm Lambda
Frontal	200, 100, 100	1.96 W × 0.98 D	− 330 to − 130 vx; − 3.20 to − 1.26 mm	− 200 to − 100 vx; − 1.96 to − 0.97 mm Bregma
Extended Interparietal (Supp. Fig. 1)	700, 150, 150	6.86 W × 1.47 D	− 350 to + 350 vx; − 3.39 to + 3.39 mm	+ 100 to + 250 vx; + 0.97 to + 2.43 mm Lambda

Analysis of mean bone thickness along the rostrocaudal axis was performed using the BoneJ2 Slice Geometry tool. In order to validate the span of interparietal bone included in the primary analyses, the Slice Geometry tool was also used to interrogate mean thickness along the sagittal axis in extended interparietal ROIs consisting of 700 images, each measuring 150 vx deep.

The diploë component of interparietal and frontal bone ROIs was further investigated to quantify the marrow cavity volume of each sample. The diploë were isolated from interparietal and frontal cortical bone layers by limiting the sample height to 50 vx and 30 vx, respectively. The ImageJ plugin 3D Objects Counter^33^ was then used to measure the frequency and volume of marrow cavities (i.e. void volume).

Both the left and right parietal bone from each bone sample was analyzed. As no differences were found in any measured parameters when comparing the left and right parietal bone regions, the left side only has been reported here for clarity. Frontal bone was only available for analysis in the 10 week samples, and the left side only was analyzed. One frontal bone sample was excluded due to the scan being of inappropriate resolution for analysis.

### Histology

Decalcification, sectioning and staining of bone samples was performed by the Monash Histology Platform (Alfred Research Alliance Node, Prahran, VIC, Australia). A 10% ethylenediaminetetraacetic acid (EDTA) solution (pH 7.4) was prepared and used to decalcify bone samples. A 2–3 week period was required to ensure complete decalcification, with EDTA solution being changed weekly. Samples were processed with Leica Peloris II tissue processor and then embedded in paraffin molds for microtomy using a Leica HistoCore BIOCUT microtome (Leica Microsystems, Wetzlar, Germany). ROIs for the left parietal and interparietal bone were defined relative to the Lambda suture. Five µm sections were collected at intervals of 150 µm in the range of − 3.5 to − 2.0 mm (parietal) and + 1.0 mm to + 2.5 mm (interparietal) for histological investigation. Standard hematoxylin and eosin (H&E) staining was performed on two sections per ROI using a Leica Autostainer XL. In brief, deparaffinized and rehydrated sections were incubated in Harris’s Hematoxylin for 5 min, rinsed and then dipped in acid alcohol solution. Following further rinsing, sections were incubated in eosin solution for 5 min, then processed for coverslipping.

### Statistical analysis

MicroCT data were compiled and calculated using Microsoft Excel. Statistical tests were performed with GraphPad Prism software v. 9.0 (GraphPad Software Inc., La Jolla, CA). Data were tested for normal distribution and passed the Shapiro–Wilk and/or Kolmogorov–Smirnov test for normality. One-way analysis of variance (ANOVA) was used to evaluate differences between the four experimental groups at the 2 week time point. Tukey’s post-hoc tests were assessed when a significant main effect was detected by ANOVA. Unpaired t-tests were used to compare the sham and 2× mTBI group at the 10 week time point. Values are expressed as group mean ± SEM. Statistical significance was defined as *p* < 0.05, with *p* values, R^2^ effect sizes and F statistics reported to 2 decimal places. For all graphical representation of data, both individual (grey dots) and mean group values (white and black bars) ± SEM are presented.

## Results

### Proximal to the impact site, the interparietal bone shows increased thickness over time after repeated mTBI

**Figure 2:**
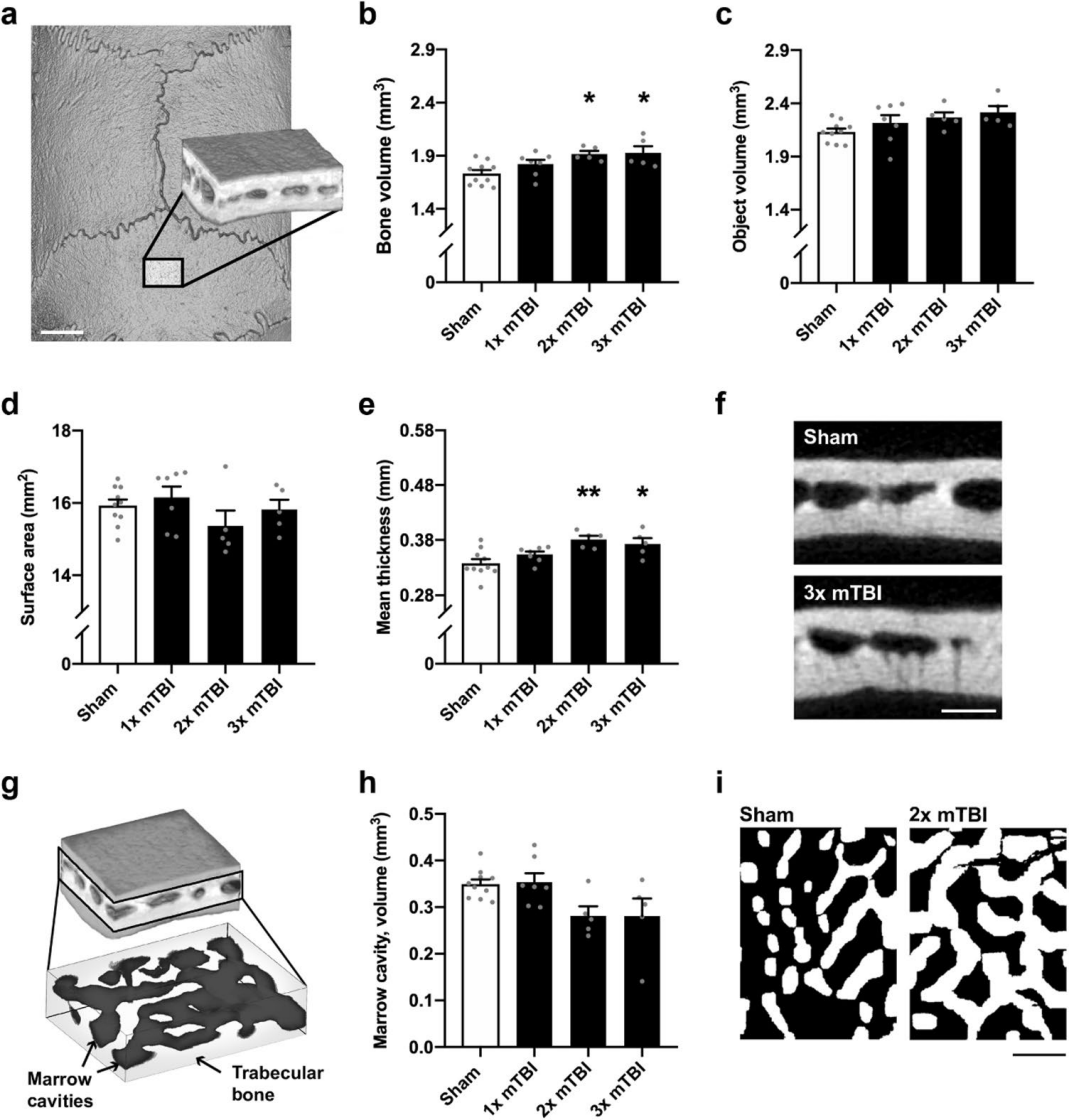
Modest increases in bone volume and thickness of the interparietal bone two weeks after rmTBI. The interparietal bone region-of-interest (ROI) is depicted in (**a**), which consisted of 150 microCT images sampled at the midline, caudal to the Lambda suture margin (scale bar = 2 mm). Quantification of mineralized bone volume (**b**) and mineralized bone thickness (e) revealed an increase in 2× and 3× mTBI mice compared to sham controls (post-hoc, *p < 0.05, **p < 0.005); whereas no significant differences were observed between groups in total object volume (**c**) or surface area (**d**). Representative Sham and 2× mTBI greyscale microCT images (**f**) illustrate modest bone thickening with rmTBI (scale bar = 500 µm). In (**g**), bone marrow cavities (dark grey) were isolated from trabecular bone (light grey) in the diploë region of the interparietal bone scan ROIs for volumetric analysis (**h**), revealing a reduction in marrow cavity volume (one-way ANOVA p = 0.02; post-hoc n.s.). Representative superior-view, binary projections of the diploë (**i**) illustrate a subtle reduction in marrow cavities with injury (scale bar = 500 µm; black indicates marrow cavity and white indicates bone). N = 5–10/group; one-way ANOVAs with Tukey’s post-hoc as appropriate.

The midline interparietal bone was firstly examined by microCT to assess the potential effects of a single or repeated mTBI to the young adult rat. At 2 weeks post-injury (Fig. 2a, b), mTBI had a subtle but significant anabolic effect, indicated by a greater bone volume (F_3,23_ = 5.20, *p* = 0.01, R^2^ = 0.40) in the 2× and 3× mTBI groups compared to the sham controls (Tukey’s post-hoc, *p* < 0.02). A slight increase in total object volume (i.e. comprising bone and marrow space) was also observed after TBI, but this did not reach statistical significance (Fig. 2c; F_3,23_ = 2.39, *p* = 0.10, R^2^ = 0.24); whereas external surface area was not different between groups (Fig. 2d, F_3,23_ = 1.26, *p* = 0.31, R^2^ = 0.14). Mean bone thickness (Fig. 2e, f) was the main contributor to the increase in bone volume, with an increase after TBI, in both the 2× and 3× mTBI groups (F_3,23_ = 6.33, *p* = 0.003, R^2^ = 0.45; post-hoc *p* < 0.005 and *p* < 0.05, respectively).

Repeated mTBI also appeared to reduce the total volume of bone marrow cavities in the diploë region at 2 weeks post-injury (Fig. 2g–i), with a significant effect of injury detected by one-way ANOVA (F_3,23_ = 3.91, *p* = 0.02, R^2^ = 0.34), but post-hoc analysis did not reveal a significant reduction in the 2× mTBI and 3× mTBI groups compared to sham controls (post-hoc; *p* = 0.07 and *p* = 0.06, respectively).

**Figure 3:**
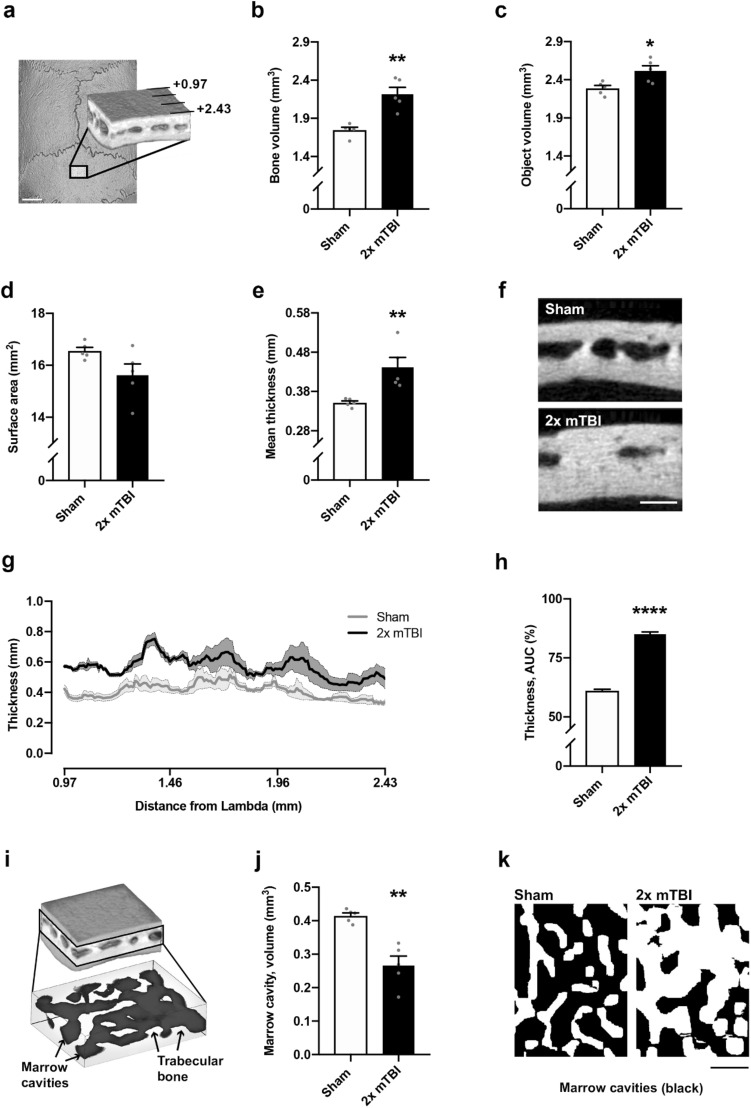
A robust increase in volume and thickness of the interparietal bone was observed at 10 weeks after rmTBI. The interparietal bone region-of-interest (ROI) is depicted in (**a**), mirroring the ROI considered at the 2 week time point (scale bar = 2 mm). Quantification of mineralized bone volume (**b**) revealed a significant increase in the 2× mTBI group compared to sham controls (t-test, **p < 0.01), as well as the object volume (i.e. bone plus marrow cavity space; **c**; *p = 0.02). Exterior surface area did not reach statistical significance (**d**). Mean mineralized bone thickness, averaged across 150 adjacent sections, was significantly increased in 2× mTBI mice (**e**, post-hoc **p < 0.001), as visualized by representative greyscale microCT images from the ROI z-stack center (**f**, scale bar = 500 µm). Mineralized bone thickness was also visualized across the rostrocaudal axis (**g**), with the 2× mTBI group (black line, dark grey error range) across the 150-image span of the 1.47 mm deep interparietal ROI, which was quantified by area under curve analyses (**h**, ****p < 0.0001). The volume of marrow cavities (dark grey) were distinguished from trabecular bone (light grey) in the diploë region of the interparietal bone (**i**), revealing a significant decrease in total cavity volume in the 2× mTBI group compared to sham controls (**j**, **p < 0.01). Representative superior view, binary projections of the diploë (**k**) illustrates a robust reduction in marrow cavities in 2× mTBI mice compared with sham controls (scale bar = 500 µm). N = 5/group; unpaired student’s t-tests.

At 10 weeks post-injury, these anabolic effects on the interparietal bone were more pronounced. Specifically, we observed a greater bone volume (Fig. 3a, b; t_8_ = 4.88, *p* < 0.01, R^2^ = 0.75) and total object volume (bone plus marrow cavity space; Fig. 3c; t_8_ = 3.00, *p* = 0.02, R^2^ = 0.53) in 2× mTBI mice compared to sham controls, but no difference in the surface area between groups (Fig. 3d; t_8_ = 2.04, *p* = 0.08, R^2^ = 0.34).

Mean bone thickness at 10 weeks post-injury, when averaged across all 150 adjacent microCT sections (Fig. 3e, f; t_8_ = 3.50, *p* = 0.01, R^2^ = 0.61), was significantly greater in 2 × mTBI mice compared to sham (**p < 0.001). To evaluate whether this effect varied across the rostrocaudal axis, bone thickness was also depicted across the ROI cross-section, which was quantified by area under the curve analysis to confirm a significant increase in bone thickness in the 2 × mTBI group compared to sham (Fig. 3g, h; t_1600_ = 21.75, *p* < 0.0001, R^2^ = 0.18). Evaluation of interparietal bone thickness across a sagittal cross-section demonstrated that this effect was most prominent close to the midline and site of TBI impact (Suppl. Fig. 1).

Also at 10 weeks post-injury, the volume of bone marrow cavities was significantly reduced in 2× mTBI mice compared to sham controls (Fig. 3i–k; t_8_ = 5.02, *p* < 0.01, R^2^ = 0.76), with this quantified difference being clearly evident upon visual inspection of representative images. Together, these findings indicate greater interparietal diploë trabecular (strut) bone volume and thickness, and a corresponding reduction in marrow cavities that progresses from 2 to 10 weeks post-injury in mice that sustained 2× mTBI insults.

**Figure 4:**
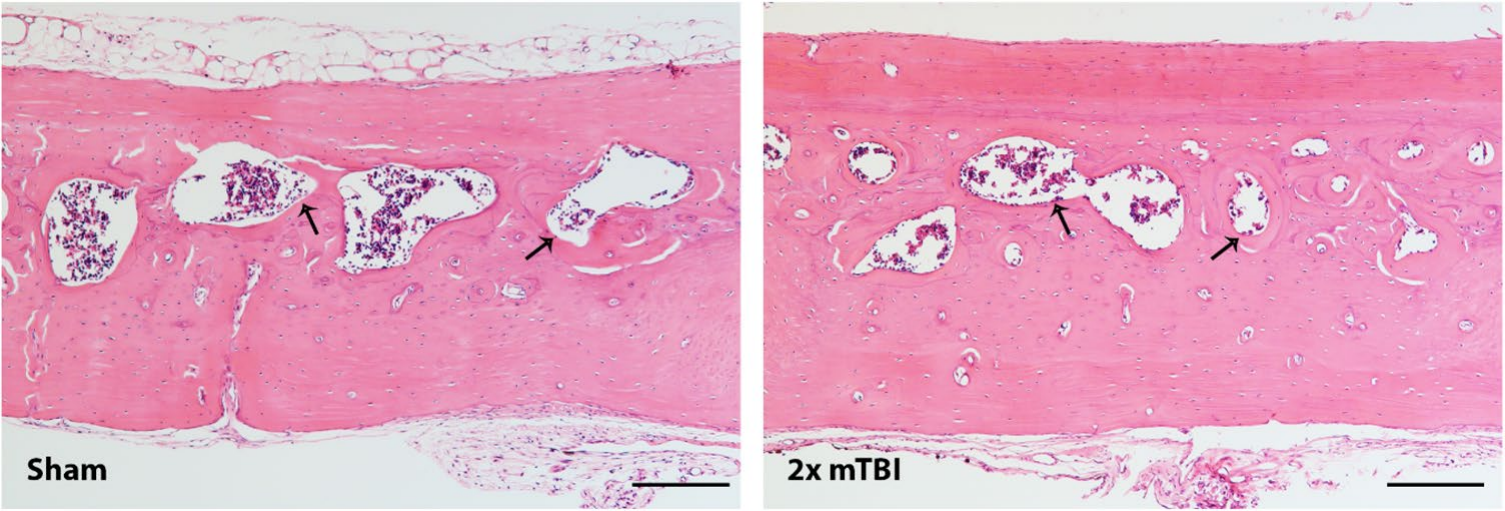
Hematoxylin and eosin staining of representative interparietal skull bone samples from sham and 2 × mTBI rats, at 10 weeks post-injury. A larger volume of trabecular bone is evident in the 2 × mTBI sample compared to sham, alongside a reduction in the volume of marrow cavities (arrows). Scale bar = 200 µm.

These TBI-induced changes in skull bone thickness and the reduction in marrow cavities within the diploë region were also apparent by H&E staining of decalcified bone sections (Fig. 4), whereas no overt differences were observed between H&E-stained bone of sham and rmTBI animals when collected at the 2 week time point (not shown).

### Distal to the impact site, the parietal and frontal bones were largely unaffected by repeated mTBI

**Figure 5:**
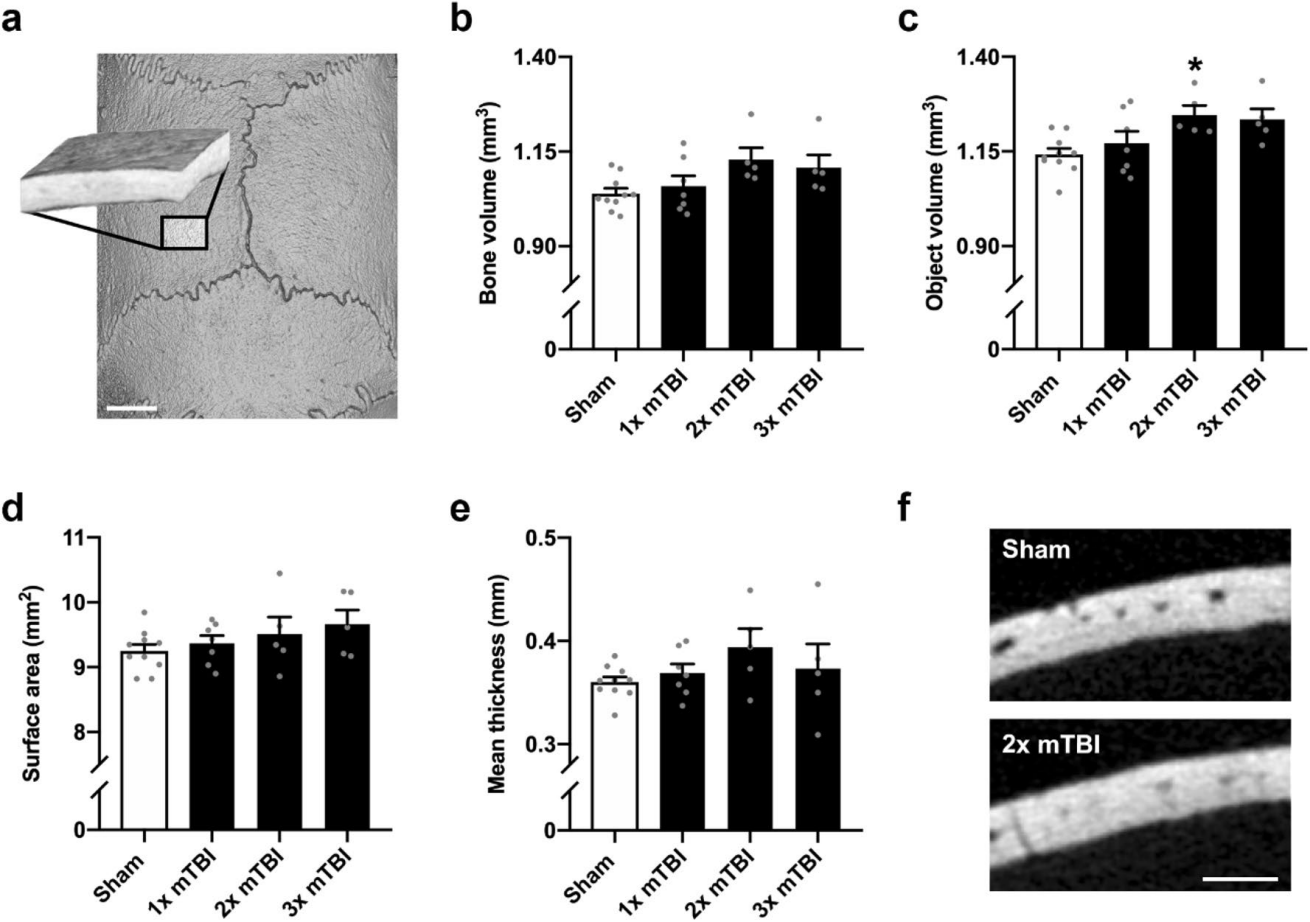
No changes in bone volume, surface area or thickness of the parietal bone were observed at 2 weeks after rmTBI. The parietal bone region-of-interest (ROI) consisting of 150 microCT images (**a**) was sampled from the mediocaudal left parietal bone, adjacent to the midline and Lambda suture margins (scale bar = 2 mm). Neither single nor repeated mTBI influenced bone volume (**b**, p = 0.23) or surface area (**d**, p = 0.35). Mean bone thickness was also not affected by injury (**e**, p = 0.58), as demonstrated by representative greyscale microCT images from the ROI z-stack center (**f**, scale bar = 500 µm). Total object volume was slightly increased in the 2 × mTBI group (**c**, *p < 0.05). Parietal ROIs from both hemispheres were examined but showed similar findings, hence only the left ROI is presented. N = 5–10/group; one-way ANOVAs with Tukey’s post-hoc as appropriate.

We next examined the mid-parietal bone by microCT (Fig. 5a), as a region more distal from the injury impact site. At 2 weeks post-injury, there was no significant effect of injury on bone volume (Fig. 5b; F_3,23_ = 2.95, *p* = 0.054, R^2^ = 0.28) or exterior surface area (Fig. 5d; F_3,23_ = 1.26, *p* = 0.31, R^2^ = 0.14). In addition, bone thickness was not affected by injury at this site (Fig. 5e, f; F_3,23_ = 1.28, *p* = 0.31, R^2^ = 0.14). A small increase in total object volume was observed in the 2× mTBI group (Fig. 5c; F_3,23_ = 4.22, *p* = 0.02, R^2^ = 0.36; post-hoc *p* < 0.05).

**Figure 6:**
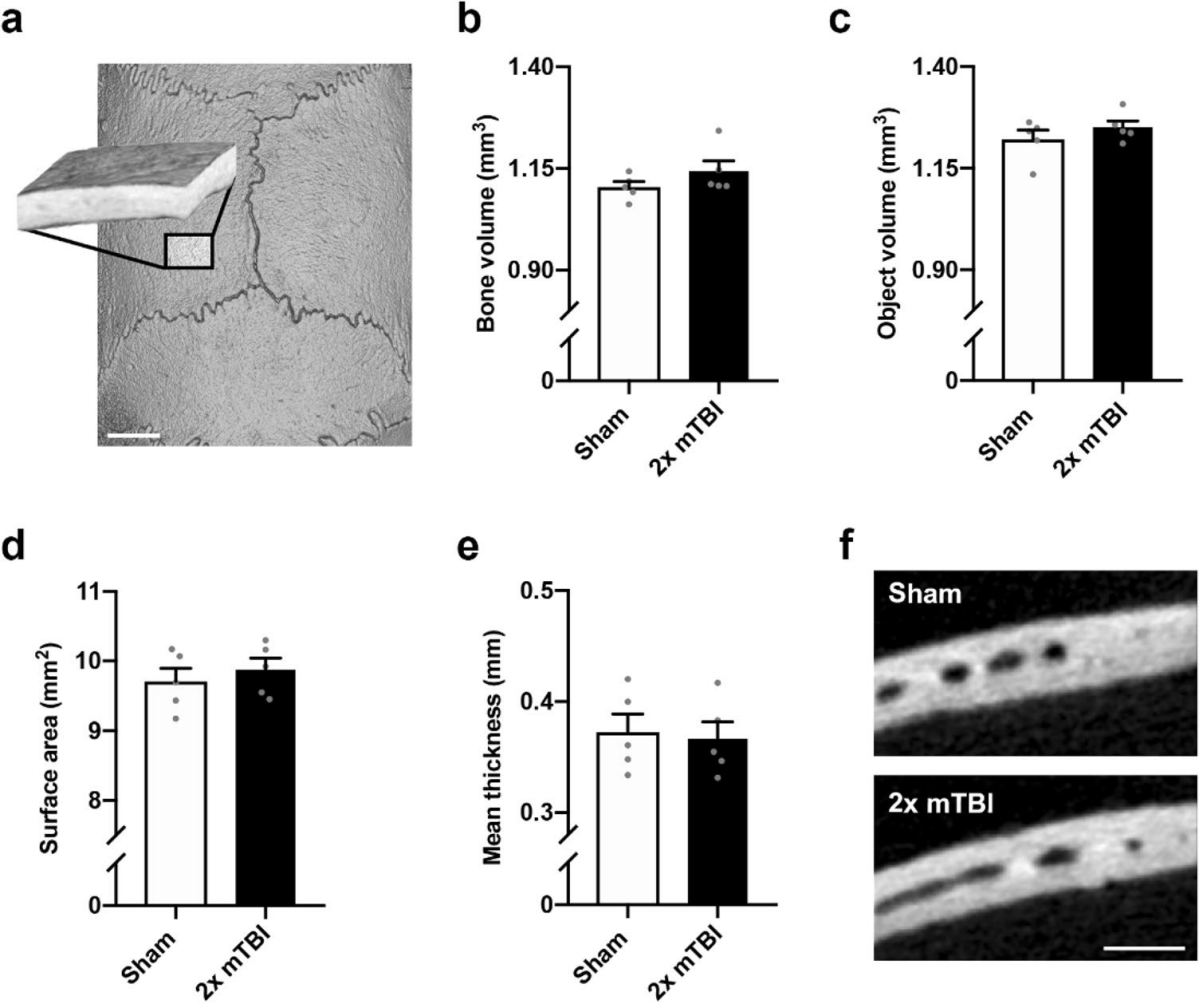
No change in volume, surface area or thickness of the parietal bone at 10 weeks after rmTBI. The parietal bone region-of-interest (ROI) consisting of 150 microCT images (**a**) was sampled from the mediocaudal left parietal bone, adjacent to the midline and Lambda suture margins (scale bar = 2 mm). Neither single nor repeated mTBI influenced mineralized bone volume (**b**, p = 0.22), total object volume (**c**, p = 0.32), or exterior surface area (**d**, p = 0.52). Mean mineralized bone thickness was also not affected by injury (**e**, p = 0.80), as demonstrated by representative greyscale microCT images from the ROI z-stack center (**f**, scale bar = 500 µm). Parietal ROIs from both hemispheres were examined but showed comparable findings; hence only the left ROI is presented. N = 5–10/group; one-way ANOVA.

At 10 weeks post-injury, the left parietal bone was similarly unaffected by repeated mild TBI, with no differences between sham and 2 × mTBI groups in mineralized bone volume (Fig. 6a, b; t_8_ = 1.33, *p* = 0.22, R^2^ = 0.18), total object volume (Fig. 6c; t_8_ = 1.05, *p* = 0.32, R^2^ = 0.12), or exterior surface area (Fig. 6d; t_8_ = 0.68, *p* = 0.52, R^2^ = 0.05). Further, bone thickness was also not affected by injury (Fig. 6e, f; t_8_ = 0.27, *p* = 0.80, R^2^ = 0.01).

Finally, no differences in any quantified parameters (mineralized bone volume, object volume (bone plus marrow cavity space), exterior surface area, bone thickness, or marrow cavity volume) were observed in the left frontal bone at 10 weeks post-injury, even more remote to the impact site, in 2× mTBI samples compared to Sham controls (Suppl. Fig. 2).

## Discussion

In this study, we examined whether mild closed-head injuries to the female rat skull—up to three impacts, spaced 24 h apart—would result in changes within cranial bone detectable by microCT and histology. In this model of mTBI, we have previously reported that 2× mTBI animals showed modest cognitive memory deficits, increased microglial reactivity, lipid peroxidation and reduced white matter myelin integrity compared to sham controls and a single mTBI insult. Increasing the number of impacts beyond two did not necessarily exacerbate these effects. Indeed, with 3× mTBI, no effects on acute behavioral function, lipid peroxidation or chronic microglia number or reactivity were observed^22,23^. Now, we have extended our characterization of this model to reveal that rmTBI also modifies the impacted skull bone in a time- and location-dependent manner. Specifically, a localized increase in cranial bone thickness and volume, coincidental with a reduction in marrow cavity volume, appeared to evolve from 2 to 10 weeks, and was largely restricted to the interparietal bone in close proximity to the impact location. These findings confirm our previous pilot work suggesting that a single mTBI over the unilateral parietal bone in adolescent male mice led to a localized increase in bone thickness after several weeks^11^, and indicate that this phenomenon can be observed across different species, experimental TBI models, and injury locations.

Our rat model of RmTBI involved repeated insults at 24 h intervals, in line with evidence from previous studies reporting a period of vulnerability of up to 3 days, whereby a repeated injury during this window has additive effects on cognition, neurodegeneration and neuroinflammation^34^. It remains to be seen how different inter-injury intervals, differing injury severities, and even the age at which an injury is sustained, may influence cranial bone responses to RmTBI. One limitation of our study design was the lack of a 3× RmTBI group at the 10 week time point, with future studies required to determine whether our observations are dependent upon the number of repeated injuries in the chronic post-injury phase.

The most likely mechanism underlying the observed cranial changes is mechanical force, in alignment with bone structure being altered in close proximity to the impact site, but not in more remote regions of the cranium. While mTBI or concussive head impacts in isolation typically involve mechanical forces below the threshold required to induce a skull fracture, mechanical loading to the cranium may nonetheless result in subtle and transient deformation of the skull, stimulating a localized reparative response characterized by enhanced bone formation by osteoblasts^35^. Indeed, a large body of work has demonstrated this phenomenon in the context of tibial compression, where mechanical loading stimulates increased cortical and trabecular bone formation^36^. Future studies incorporating sophisticated techniques to label and track the response and activity of osteoblasts and osteoclasts will be required to define the precise mechanisms involved. It is plausible, for example, that changes in osteocyte production of the mechanosensitive Wnt inhibitor sclerostin is involved in this response^37–39^.

Although there is a scarcity of published literature on skull responses after mild head impacts, cranial bone thickening has previously been reported in the context of hydrocephalus, raising the intriguing possibility that abnormal pressure from cerebrospinal fluid build-up or swelling—as can occur after a TBI—may also act as an intracranial mechanical regulator of bone growth^40^. Alternatively, as most of these hydrocephalus patients had ventricular shunts placed during early childhood resulting in chronic intracranial hypotension, the subsequent lack of outward pressure on the developing skull may result in bone thickening to fill the vacant space^41–43^. It is unclear whether similar interactions might occur between the cranium and cerebrospinal fluid in the context of TBI, particularly after mTBI in which significant swelling or changes in intracranial pressure are unlikely. However, these case studies do highlight the potential of skull bone to adapt and respond to external stimuli.

The implications of our findings—that rmTBI resulted in localized thickening of the skull by 10 weeks post-injury—may prove controversial. Cranial bone thickness and strength is developmentally-dependent^44^, and skull thickness is an important determinant of the propensity of the skull to deform and/or fracture^45,46^. Even in adulthood, experimental closed head impacts to middle-aged (20 week old) mice result in a lower incidence of skull fracture compared to similar injuries in younger mice (10 weeks old), coinciding with the older animals having thicker skulls^10^. Across a longer time course, cortical thinning, a key component of fracture risk, is associated with aging in most of the skeletal system, including the skull^47^. Our findings raise the interesting question of whether increased skull thickness after repeated mTBI has consequences for the strength of the bone, and how a subsequent force is transduced. The mechanical strength of cranial bones was not evaluated in this study; such an investigation would require the development of validated methods, and would be a useful addition for future studies to determine the functional consequences of observed changes in bone thickness.

Theoretically, thicker cranial bone would translate to any subsequent head impacts being transduced to a lesser extent than in a skull with no prior impacts. Indeed, we observed this previously in the mouse; a prior mTBI at postnatal day 35 reduced the incidence of skull fracture after a second mTBI induced at postnatal day 70^11^. One might surmise from this logic that mild impacts to the skull, below the threshold required to induce a skull fracture, might be protective, in that they promote bone growth to provide greater protection against future injuries—a potentially useful phenomenon in contact sports and military environments in which there is a risk of repeated head impacts. However, we are certainly not advocating for voluntarily acquiring repeated head impacts as a form of preconditioning! Accumulating evidence continues to demonstrate that repeated exposure to head impacts is detrimental to brain structure and function, as well as recovery after subsequent hits^48–51^. Rather, our findings highlight the need for studies to consider how the whole head is impacted by a mTBI or concussion, including the skull—as well as connective tissue, meninges, skin and vasculature; and to better understand the complex brain-bone relationship under conditions of repetitive mild mechanical loading. For example, it is plausible that cranial bone responsiveness to mechanical trauma interacts with changes in the composition and function of the associated dura. This could have implications for the neuroinflammatory response in the underlying brain parenchyma. It has also been suggested that bone remodeling in this context might be involved in the emergence of cerebrospinal fluid leakage, a relatively rare occurrence after closed head injuries associated with a risk of life-threatening meningitis^52,53^. However, our data did not reveal any opening of fracture lines, and we suggest that this would require greater mechanical force than what we have studied here.

## Conclusion

We herein report greater localized cranial thickness and lowered calvarial porosity after rmTBI in the female rat, which are both time- and location-dependent. While the consequences of such changes on the mechanical properties of the skull remain to be elucidated, our findings support future studies adopting a more holistic investigation of the mechanisms and consequences of repeated head impacts on the skull as well as the brain tissue itself.

## Supplementary Information

Supplementary Information.

## Data Availability

The datasets generated during and/or analyzed during the current study are available from the corresponding author on reasonable request.
